# Coverage of Cervical Cancer Screening in Catalonia for the Period 2008–2011 among Immigrants and Spanish-Born Women

**DOI:** 10.3389/fonc.2013.00297

**Published:** 2013-12-20

**Authors:** Vanesa Rodríguez-Salés, Esther Roura, Raquel Ibañez, Mercè Peris, F. Xavier Bosch, Sílvia de Sanjosé

**Affiliations:** ^1^Unit of Infections and Cancer, Cancer Epidemiology Research Programme, Catalan Institute of Oncology, IDIBELL, Barcelona, Spain; ^2^CIBER en Epidemiología y Salud Pública (CIBERESP), Barcelona, Spain

**Keywords:** cytology, cervical cancer screening, immigrant, coverage, health services

## Abstract

**Background:** Female immigration in Catalonia, Spain, increased dramatically in the last 10 years. The Public Health system in the Region, provides a free of charge opportunistic cervical cancer screening.

**Aim:** This study examines cervical cancer screening coverage and prevalence of cytology abnormalities in Catalonia by immigration status.

**Methods:** The study analyzes the cytologies registered among women aged 25–65 that have been attended at the Primary Health Centers (PHC) for any reason (*n* = 1,242,230) during 2008–2011. Coverage was estimated from Governmental data base Information System Primary Care (SISAP) that includes 77% of PHC. The database is anonymous, and includes information on age, country of birth, diagnostic center, and cytology results.

**Results:** During the period 2008–2011, 642,643 smears were performed in a total of 506,189 women over 14 years, of whom 18.3% were immigrants. Cytology coverage was higher among immigrant women compared to Spanish born (51.2 and 39% respectively). Immigrant women also had a higher prevalence of abnormal Paps compared to the Spanish population, 4.5 and 2.9% respectively (*p* < 0.001).

**Conclusion:** Immigrant women in Catalonia had a high access to the Public Health Services and to cervical cancer screening facilities. The higher prevalence of abnormal cytologies in immigrant women compared to native women indicates the relevance to prioritize cervical cancer screening activities on a regular base in new comers.

## Introduction

In 2011 in Catalonia, a region in the North-East part of Spain, 15.7% of the population had been born outside Spain (1). The migration growth had been steady for the last 10 years at 1.2 per 1,000 inhabitants. Latin America and Northern-African countries were the most common regions of migration origin (2).

Cervical cancer is the third most common cancer in women. More than 85% of the global burden occurs in developing countries (3). Among all malignant tumors, cervical cancer is the one which can be most effectively controlled by screening (4). In high-income countries, the incidence is generally lower because of the availability of cervical screening. Based on the country of origin as a reflection of potential contrasting cultures with the reception country, migrants may have particular characteristics that pose them to a differential risk when compared to native population in the destination country. While migrants may benefit of a better access to qualified health attention they may also have higher risk for certain diseases. Azerkan et al. (5) have been reporting that migrant population in Sweden largely lowered their risk of cervical cancer as compared to the country of origin but had a lower coverage of cervical cancer screening coverage. However, they identified an important variability in relation to the country of origin (5).

In Catalonia in 2007, with an opportunistic screening, the annual incidence rate of cervical cancer was 7 per 10^5^ women and the mortality rate was 1.2 per 10^5^ women. The annual percentage change of mortality during the period 1993–2007 was −4.0 (6, 7).

The aim of this study was to examine cervical cancer screening coverage and prevalence of cytology abnormalities by immigration status among women attended within the Public Sector of Catalonia during 2008–2011.

## Materials and Methods

The Catalan Health Care System is a public facility providing a universal coverage guaranteed by the National Health Service (CatSalut) integrating into a single network of public use all health and social resources. The principle supplier of this system is the Catalan Institute of Health (CIH) which provides services to about 75% of the population. It is estimated that around 30% of the population has double health insurance, public and private (8, 9).

In Catalonia the cervical cancer screening is opportunistic. The latest recommendations on screening protocols for cervical cancer were introduced in 2006. Special emphasis was directed to increase coverage among the under screened women above age 40 and to reach a 3-year interval between cytologies for the target population 25–65 years old (10). During the study period, foreigners residing in Spain who were registered at the city council had the same rights to health care as Spanish nationals, regardless of their residence status (11, 12).

### Study population

The study population consisted of all Spanish or immigrants women living in Catalonia with a registered age of 25–65 years old. Information on cytology(ies) performed at basic health areas within the public health system were obtained for the study period 2008–2011. Data were extracted from the routine data base System of Primary Care (SISAP) which is used in 77% of the public basic health areas (13). The available information consisted of place and date of birth, collection of cytology and results, and the health center identification. For this study immigrant population was considered any person whose place of birth was registered as being outside the Spanish territory.

Age was categorized in two groups: 25–39 and 40–65. Cytology results were categorized following the Bethesda system (12). The result of HSIL+ (including HSIL and suspected of cancer) was analyzed individually and by country of origin, using the following 16 groups: Spanish-born (native women), Oceania, Caribbean, Central America, Eastern Asia, Eastern Europe, North America, Northern Africa, Northern Europe, South America, South-Eastern Asia, South-Central Asia, Southern Europe (without Spain), Sub-Saharan Africa, Western Asia, and Western Europe (14).

Cytology results refer to Bethesda classification that classifies as negative, atypical squamous cells of undetermined significance (ASCUS), atypical glandular cells of undetermined significance (AGUS), low grade intraepithelial lesions (LSIL), high grade intraepithelial lesions (HSIL), and suspected carcinoma. Whenever we refer to HSIL+ we include HSIL and suspicion of cancer.

### Statistical analysis

The results for continuous variables are given as mean values and SD, and for categorical variables as percentages. We used the chi-square test to compare proportion between groups and Student’s *t*-test to compare continuous variables. To estimate the coverage of cervical cancer screening, cytologies were related to the eligible population to provide a sense of the impact in the general population. Further, cytologies were also related to women that had been attended in any of the primary health care center for any reason. To estimate the coverage of cervical cancer screening, number of women with at least one cytology in this period 2008–2011 was related to either all eligible women or to those attended at the primary health care facilities during the study period. Estimates are provided by geographical region of origin. Estimates are given as percentage with 95% confidence intervals (CI). The interval between two cytologies was estimated by selecting all women that between January and December 2008 had a cytology with a negative result and following them until December 2011 for a second cytology within this period. A lineal regression model between coverage and use of health services was constructed. *p*-Values smaller than 0.05 were considered to be statistically significant.

Data analyses were conducted using R (R Foundation for Statistical Computing, Vienna, Austria; Version 2.14.0).

## Results

**Figure 1:**
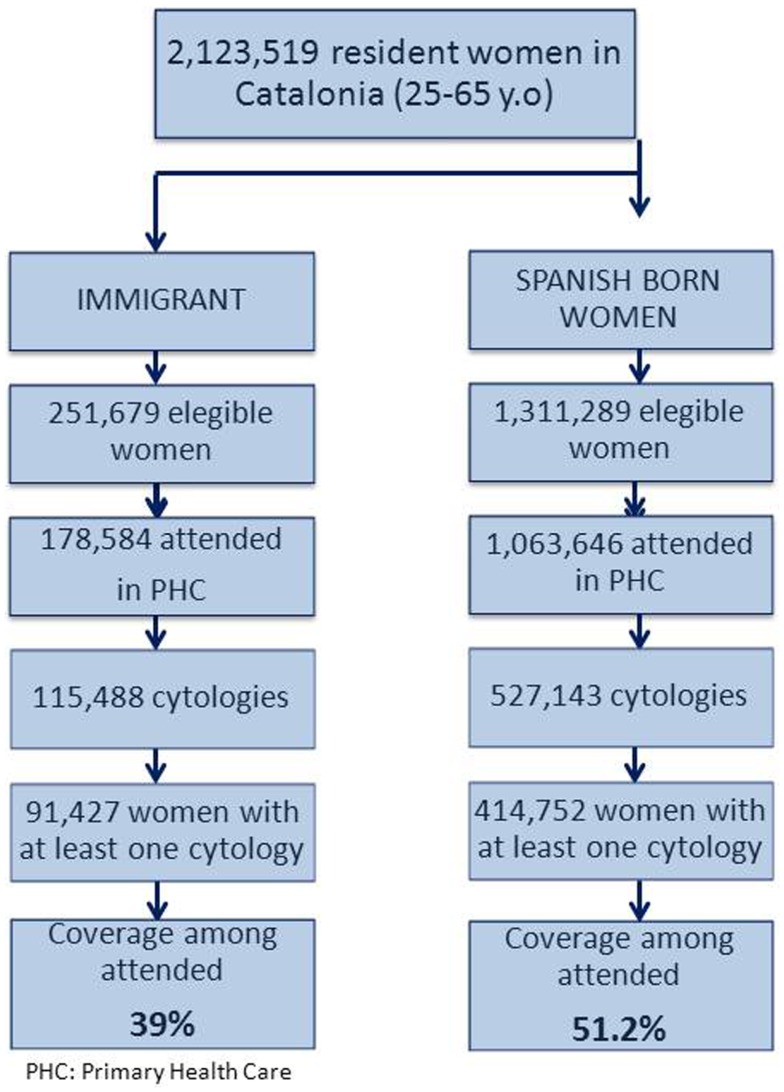
**Flow chart describing the inclusion of participants in the study**.

Figure 1 shows a flow chart that describes the inclusion of participants in the study. Table 1 shows the characteristics of the study population by immigration status for the period 2008–2011. A total of 642,643 cytologies were performed in a total of 506,189 women 25–65 years old. Of them, 91,427 (18%) were immigrant. Women from Morocco, Ecuador, Bolivia, Colombia, and Romania represented 51% of all immigrants (data not shown). Spanish-born women were significantly older than immigrants (mean age = 44.3 and 36.3 years respectively, *p*-value < 0.001). The ratio of cytology per woman was similar in the two groups and this was irrespective of cytology results. However the ratio of cytology coverage among attended women by migration status and age strata showed that younger immigrant women (<40) had higher coverage compared to their elder women (>39) with a ratio of 1.26 while among Spanish women the ratio was 1.07. Cytology coverage was statistically higher among immigrants (51%) as compared to Spanish-born women (39%). Similarly, immigrant women were more likely to have an abnormal cytology compare to Spanish born (4.5 versus 2.9%, *p*-value < 0.001). Participation to second round screening among women with a normal cytology was lower among immigrant women (43.1%) as compared to Spanish women (50.7%).

**Table 1:** **Characteristics of women aged 25–65 years old included in the evaluation of cervical cancer screening coverage in Catalonia during the period 2008–2011 by immigration status**.

	Population 25–65 years old			
	Immigrant^a^	Spanish born	*p*-Value*	All population^b^
	(*n* = 91,427) *N* (%)	(*n* = 414,752) *N* (%)		(*n* = 506,189) *N* (%)
No. of cytologies	115,488	527,143		642,643
No. of women with cytology	91,427	414,752	<0.001	506,189
Age, years (SD)	36.3 (8.2)	44.3 (11.0)	<0.001	42.8 (11.0)
Ratio of cytology/women	1.26	1.27		1.27
Cytology coverage, % (CI 95%)
Eligible women in the target population
25–39 years old	38.6 (38.3–38.8)	30.9 (30.8–31.1)		32.8 (32.7–32.9)
40–65 years old	32.0 (31.7–32.3)	32.1 (32.0–32.2)		32.1 (32.0–32.2)
25–65 years old	36.4 (36.2–36.5)	31.6 (31.5–31.7)		32.4 (32.3–32.5)
Attended women in primary health care
25–39 years old	55.3 (55–55.6)	40.7 (40.5–40.8)		44.0 (43.8–44.1)
40–65 years old	43.6 (43.2–44)	38.0 (37.9–38.1)		38.5 (38.4–38.6)
25–65 years old	51.2 (51–51.5)	39.0 (38.9–39.1)		40.8 (40.7–40.8)
Cytology results			<0.001	
Number of women with positive cytology	4,072 (4.5)	12,190 (2.9)		16,262 (3.2)
Number of women with negative cytology	88,948 (97.3)	408,041 (98.4)		496,999 (98.2)
Compliance of second round of screening when the first cytology was negative in 2008, %	43.1	50.7		50.2

*No., number; SD, standard deviation; CI, confidence interval*.

^a^ Refers to those with place of birth was outside the Spanish territory. *p-Value was calculated using chi-square test to compare categorical variables and Student’s *t*-test to compare continuous variables.

*^b^ Include people without information of country of birth*.

Table 2 describes the immigrant population by region of origin and cytological coverage. The largest community of immigrants came from South America accounting for 51% of the total immigrants. The largest contributing country was Ecuador (13.5%). Cytology coverage was lower in South-Central Asia, Western Europe, South-Eastern Asia, and North America, while it was higher among women from Latin America and Sub-Saharan Africa. Women from Northern and Sub-Saharan Africa and South-Central Asia were more likely to use the Public Primary Health Care Services compared to those from other regions. Oceania, Eastern Asia, and North America were the groups with less use of the health services.

**Table 2:** **Region of origin of immigrant women from Catalonia by use of cervical cancer screening and primary health care services during the period 2008–2011**.

Region of origin	Eligible population	No. of women with at least one cytology (%)	Attended women	Cytology coverage in attended women, % (CI 95%)	Use of primary health care services
Europe	58,283	14,870	36,599	40.6 (40.1–41.1)	62.8
Southern Europe	8,516	2,285	5,313	43.0 (41.7–44.3)	62.4
Eastern Europe	38,507	10,084	24,554	41.1 (40.5–41.7)	63.8
Northern Europe	3,708	872	2,227	39.2 (37.1–41.2)	60.1
Western Europe	7,553	1,629	4,505	36.2 (34.8–37.6)	59.7
Africa	41,935	16,390	33,929	48.3 (47.8–48.8)	80.9
Sub-Saharan Africa	7,931	3,284	6,074	54.1 (52.8–55.3)	76.6
Northern Africa	34,004	13,106	27,855	47.1 (46.5–47.6)	81.9
Asia	20,907	5,247	12,756	41.1 (40.3–42.0)	61.0
Western Asia	2,623	741	1,623	45.7 (43.2–48.1)	61.9
Eastern Asia	11,292	2,712	5,967	45.4 (44.2–46.7)	52.8
South-Eastern Asia	1,665	428	1,102	38.8 (36.0–41.7)	66.2
South-Central Asia	5,327	1,366	4,064	33.6 (32.2–35.1)	76.3
America	130,200	54,890	95,102	57.7 (57.4–58.0)	73.0
South America	108,943	46,588	79,857	58.3 (58.0–58.7)	73.3
Central America	10,260	4,084	7,197	56.7 (55.6–57.9)	70.2
Caribbean	10,174	4,045	7,608	53.2 (52.0–54.3)	74.8
North America	823	173	440	39.3 (34.8–43.9)	53.5
Oceania	128	30	67	44.8 (32.9–56.7)	52.3

*Women aged 25–65 years old*.

Table 3 shows all cytological results by migration status performed in women aged 25–65 years for the period 2008–2011. Abnormal results were more common among immigrant women as compared to Spanish born (4.5 versus 3%, *p* < 0.001) This was explained by an increased in all categories. In particular the group of HSIL and suspected cancer were twice as common as that observed among Spanish women (*p* < 0.001). These differences were observed among women within the age group 25–39 but not among women above age 40 (Data not shown).

**Table 3:** **Cytology results for women aged 25–65 years old that participated in cervical cancer screening in Catalonia during the period 2008–2011 by immigrant status**.

Cytology results	Immigrant *N* (%)	Spanish born *N* (%)	*p*-Value*	All population^a^ *N* (%)
Negative cytology	108,940 (95.5)	506,567 (97)	<0.001	615,519
Positive cytology	5,121 (4.5)	15,902 (3)		21,023
Results of cytology
ASCUS/AGUS	2,230 (1.9)	7,472 (1.4)	<0.001	9,702 (1.5)
ASC-H	144 (0.1)	381 (0.07)	<0.001	525 (0.08)
LSIL	2,056 (1.8)	6,390 (1.2)	<0.001	8,446 (1.3)
HSIL	640 (0.6)	1,541 (0.3)	<0.001	2,181 (0.4)
Suspected cancer	51 (0.04)	118 (0.02)	<0.001	169 (0.03)
Without result of cytology	253 (0.2)	887 (0.2)		1,140 (0.2)
Not applicable	1,174 (1.2)	3,787 (0.7)		4,961 (1.0)

*p-Value was calculated using chi-square test to compare categorical variables.

*^a^ Include people without information of country of birth*.

**Figure 2:**
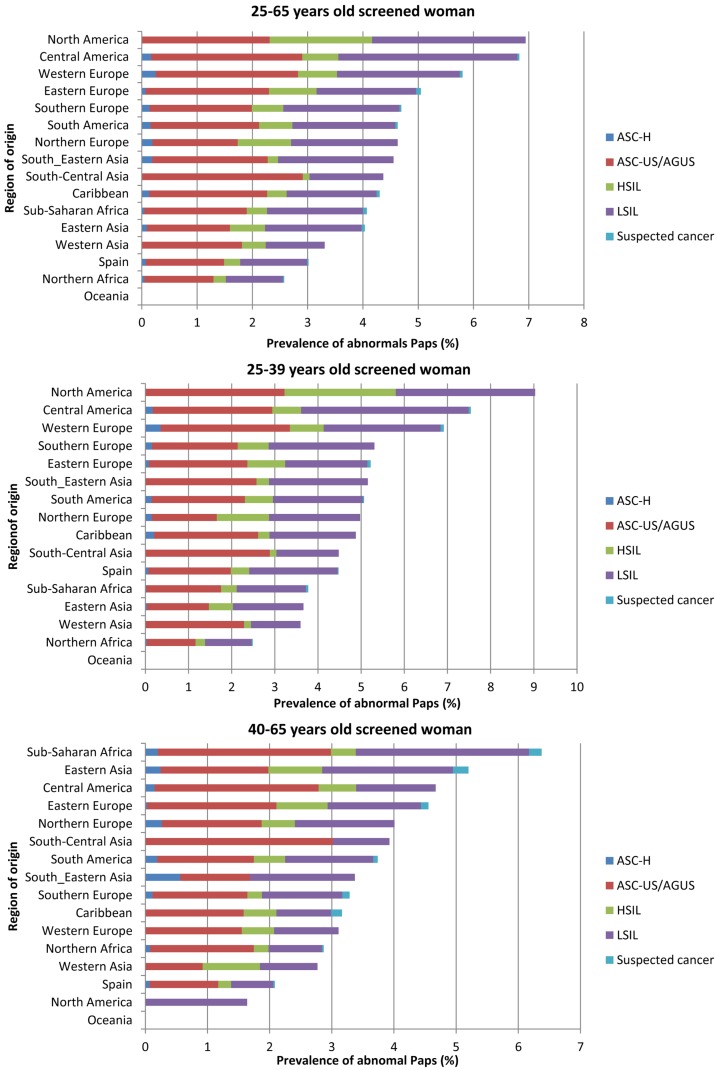
**Prevalence of abnormal cytology in woman aged 25–65 years old by region of birth**.

Figure 2 describes the distribution of abnormal diagnosis by age group and by region of origin. Interestingly, women from North America had highest percentage of abnormal cytologies (6.9%), followed by women from Central America and Western Europe (6.8 and 5.8%, respectively). In contrast, women from Oceania and Northern Africa had the lowest percentages of abnormal cytologies (0 and 2.6%, respectively). North American women mainly contributed to abnormal cytologies in the age category of 25–39 years while Sub-Saharan African women were so in the age group of 40–65 years.

**Figure 3:**
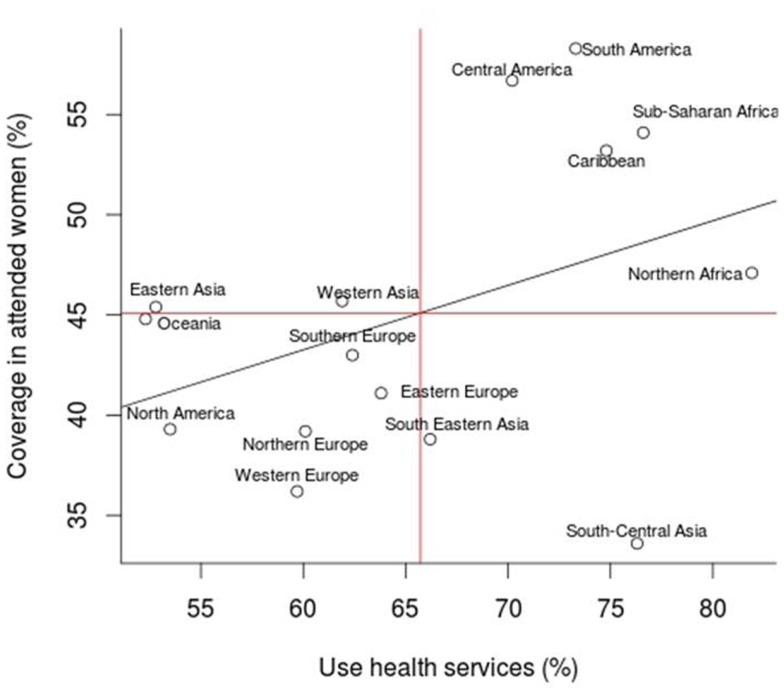
**Correlation analysis between the use of health services and the coverage of cytology by region of birth**.

Figure 3 shows the correlation between cytological coverage in attended women and use of health services in Primary Care. We found that South-Central and South-Eastern Asia were the regions that presented less coverage in attended women but that used more health services in Primary Care. In contrast, the region of Eastern Asia showed to have cytology coverage close to the average one but a lower use of health services. Despite of these differences we did not find a statistically significant correlation between cytology coverage and global use of primary health services within the Public Sector.

## Discussion

Compared to the Spanish-born women, we identified that immigrant women had a higher coverage of cervical cytology within the Public Health Sector. Among them, Latin American women showed the highest coverage (51.2%). A higher proportion of high grade intraepithelial lesions or more (HSIL+) was also observed among immigrant women, particularly among women from North and Central America.

We had previously estimated that cytology coverage in the region over a 3 years period was around 40% for the overall population irrespective of the place of birth (6). Immigrant women had a slightly higher use of Public Health Care facilities and also a higher cytological coverage than Spanish-born women. As expected, women born in economically better-off economies like Northern and Western Europe, and North America registered a lower coverage than the average. These differences could more likely be attributed to a higher use of private Health Care providers although cultural reasons cannot be dismissed. Contrary, idiomatic, cultural, and social factors are reasons more likely to explained the lower coverage observed among women from Southern Asian rather than a higher enrollment in the private sector. This is consistent with a previous study exploring cancer screening uptake in Spain were a lower use of breast and cervical cancer screening activities were reported among women language barriers like Asian countries in contrast with Latin American from Central and South America countries (15). We suggest that the language barrier could be a major limitation that explains why Latin American women had higher access to the services. This contrast with our estimates that general use of primary health services for any medical reason was higher among women born in Northern-African countries with French or Arab as main communication language. Religion and health perception may play also an important role in the decision of searching for gynecological exams among asymptomatic women within universal health coverage facilities.

An interesting fact is that Azerkan et al. (5) showed that immigrants in Sweden from North-America, Denmark, or Norway had lower participation in screening programs, as compared to immigrants from Africa or Latin American countries. These differences are indicative, along with our results, that determinants of cervical cancer screening attendance are complex. Azerkan et al. further explored the reasons why other Scandinavian countries’ women immigrating to Sweden were not participating so regularly to the screening activities as Swedish women did (5). A qualitative study identified that absence of screening was not directly related to avoidance but to work and life overload in women within the reproductive ages. These observations are also consistent with other studies (16).

On the other hand, age may be an important determinant of cytology uptake among women irrespective of the country of birth (17, 18). Overall immigrant population were younger and within the reproductive age period than Spanish born, thus age stratified analysis may better to reflect any difference in health care access. Consistently we found that women irrespective of the country of origin between 40 and 65 years old had a lower participation rate than younger women. Unfortunately we could not verify whether this observation was related to the residence period, education, or socio-economic level as observed in other studies (5, 15, 18–20).

A low percentage of immigrant women returned after the 3 years period as recommended by the screening protocol (10). Around 57% of immigrant women with a negative cytology did not return for the next screening round versus the also low, 50%, attendance observed in the Spanish-born population. We believe that this low attendance for the next screening round of both population groups is likely due to the changes in the recommendations by which women have gone from 1-year screen interval to 3-year interval. It may be easier for the women to forget about the need of a cytology unless there is a system for recall. We have observed, in a pilot study, that individual call-recall increased substantially the participation (17).

Immigrant women had a higher proportion of abnormal cytologies versus Spanish women. Over the approximately 600,000 cytologies registered during the 2008–2011 period, an 18% corresponded to immigrant women from which HSIL+ diagnosis were twice as high as that detected in Spanish-born women. These lesions were mainly detected in women 25–39 years old. On the contrary, immigrant women within the age range of 40–65 years had lower percentage of HSIL+ than native women. Women from North America had the highest prevalence of HSIL+ in all range of ages. Although based in low numbers it was surprising to observe a low cytological coverage and a high HSIL+. Both aspects could be speculated to be related to differential referral patterns. Women born in North America may have a higher socio-economic status and use more the private gynecological services. When an abnormal result is diagnosed the women may opt to attend a free of charge services.

On the other hand, women from North of Africa had a lower percentage of HSIL+ compared to Spanish-born women. These results confirm the consistently low incidence rates of cervical cancer observed in populations with Islam as religion (14).

The main strength of this study is the analysis of immigrant population evaluating the medical records of the public health system in the opportunistic screening of cervical cancer in Catalonia. Until now, most studies had used national health surveys or questionnaires for analysis of cervical cytology coverage and its determinants (8, 21). However, the use of electronic medical records eliminates the bias that exists in these types of studies. The inclusion of a large proportion of women living in Catalonia is also strength of this study.

We would have liked to evaluate our data using information on educational level, socio-economic factors, or years since immigration. These are important variables related to the participation in cervical cancer screening (5, 22). Efforts to further investigate these factors are on-going for future analyses. The available information was restricted to the one registered within the public health care system. This may underestimate the real coverage if women use both private and public systems selectively for certain specialties like gynecology. However our results on the overall cytological coverage, are aligned with those reported by personal interviews in surveys (21, 23).

Some studies have shown that immigrant population has a high mobility affecting the stability of health statistics (5). We have observed that about 5% of the immigrant population tends to change area of residence or quit the health system. We believe that this proportion is unlikely to greatly affect our results.

In summary, immigrant women in Catalonia participating in cervical cancer screening had a greater coverage and higher prevalence of cytological abnormalities, but lower participation in a second screening round versus native women. Immigrant women should be strongly recommended to participate in the screening activities on a regular base.

## Author Contributions

Conception and design of the study: Vanesa Rodríguez-Salés, Sílvia de Sanjosé. Analysis and data interpretation: Vanesa Rodríguez-Salés, Esther Roura, Sílvia de Sanjosé. Drafting the manuscript: Sílvia de Sanjosé, Vanesa Rodríguez-Salés. Critical revision of the manuscript: Esther Roura, Raquel Ibañez, Mercè Peris, F. Xavier Bosch. Final approval of the manuscript: Vanesa Rodríguez-Salés, Esther Roura, Raquel Ibañez, Mercè Peris, F. Xavier Bosch, Sílvia de Sanjosé.

## Conflict of Interest Statement

Esther Roura received occasional support for attendance at scientific conventions by either Sanofi Pasteur MSD, GlaxoSmithKline, or Qiagen. Sílvia de Sanjosé has received occasional travel fund for attendance at scientific conventions by either GlaxoSmithKline, Sanofi Pasteur MSD, Merck, or Qiagen. F. Xavier Bosch is member of the advisory board of GlaxoSmithKline, Merck Sharp & Dohme, and Sanofi Pasteur MSD and of the speakers bureau of GlaxoSmithKline. He received occasional travel fund to conferences/symposia/meetings by either GlaxoSmithKline, Sanofi Pasteur MSD, Merck & Co., or Qiagen. All other authors refer no conflict of interest.
